# Oxidized low‐density lipoproteins as a novel risk factor and therapeutic target for ACM

**DOI:** 10.15252/emmm.202114789

**Published:** 2021-08-19

**Authors:** Hoyee Tsui, Su Ji Han, Eva van Rooij

**Affiliations:** ^1^ Hubrecht Institute KNAW and University Medical Center Utrecht Utrecht The Netherlands; ^2^ Department of Cardiology University Medical Center Utrecht Utrecht University Utrecht The Netherlands

**Keywords:** Cardiovascular System

## Abstract

Arrhythmogenic cardiomyopathy (ACM) is an inherited heart disease involving arrhythmia in young adults accompanied by structural changes at later stages. In this issue of *EMBO Molecular Medicine*, Sommariva *et al* (2021) identified a positive correlation between circulating levels of oxidized low‐density lipoproteins (oxLDL) and ACM disease penetrance, which contributes to fibro‐fatty cardiac remodeling via the oxLDL/CD36/PPARγ axis. These data identify oxidized low‐density lipoproteins as a risk factor for ACM and uncover a novel therapeutic intervention option to block disease pathogenesis.

## Graphical abstract

E. van Rooij & colleagues discuss the study by Sommariva *et al*, in this issue of *EMBO Mol Med*, that identifies a positive correlation between oxLDL circulating levels and ACM disease penetrance, and uncovers a novel therapeutic intervention option to block disease pathogenesis.

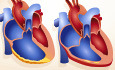

Arrhythmogenic cardiomyopathy (ACM) is a non‐curable genetic disease of the heart muscle with a prevalence of 1:1,000 to 1:5,000 (Austin *et al*, 2019). Approximately 55% of ACM patients carry a mutation in one or more of the desmosomal genes, with plakophilin‐2 (PKP2) mutations accounting for ˜40% of all ACM cases (Austin *et al*, 2019). ACM has an age‐dependent penetrance characterized by an early asymptomatic “concealed phase” between the 3^rd^ and 4^th^ decade of life, albeit with a higher propensity toward ventricular arrhythmias; ACM is responsible for > 10% of premature sudden cardiac deaths in young adults (Padrón‐Barthe *et al*, 2017; Elias Neto *et al*, 2019). With disease progression, the hallmarks at later stages include fibro‐fatty replacement of the ventricular myocardium with corresponding cardiac dysfunction and eventual heart failure in severe cases (Podgoršek *et al*, 2018).

Considerable heterogeneity is evident in ACM disease onset and manifestation, with approximately 50% of mutation carriers not developing any symptoms (van der Zwaag *et al*, 2009). Genetic predisposition alone does not necessarily dictate disease development, and other contributing factors are known to influence ACM disease onset and progression (Elias Neto *et al*, 2019). This is substantiated by clinical data showing that ACM has an age‐dependent penetrance, a male predominance with an estimated ratio of 3:1, and athletic mutation carriers have a fivefold increase in susceptibility to arrhythmias (Padrón‐Barthe *et al*, 2017; Podgoršek *et al*, 2018; Austin *et al*, 2019).

Sommariva *et al* (2021) aimed to investigate the potential role of oxidative stress in the development of ACM fibro‐fatty replacement. While oxidative stress and oxLDL have been implicated in regulating adipogenesis by increasing the expression of cluster of differentiation 36 (CD36) and peroxisome proliferator‐activated receptor gamma (PPARγ) in coronary heart diseases, its involvement in ACM is not well known (Nicholson & Hajjar, 2004).

In the current study, the authors investigated the involvement of oxidative stress in ACM by analyzing oxLDL levels from plasma samples of healthy individuals and ACM patients and found that the latter had higher circulating levels of oxLDL. Clinically, the circulating levels of oxLDL in mutation carriers showed to be of predictive value for disease manifestation, as the authors identified a positive correlation between increased oxLDL, cardiac dysfunction, and the occurrence of arrhythmic events. This study is the first to show a direct association of high levels of circulating oxLDL with disease manifestation and an increased severity in the fibro‐fatty phenotype in ACM.

Following up on their findings in patients, the authors tested the effect of oxLDL in inducing adipogenesis using cardiac‐derived mesenchymal stromal cells (C‐MSC) isolated from ACM patients and healthy controls. These cells are abundantly present in the heart and have the potential to differentiate from adipocytes (Sommariva *et al*, 2016). Post‐adipogenic induction, ACM C‐MSC demonstrated higher levels of lipid content in addition to increased expression of CD36 and PPARγ compared to controls. This effect was even more pronounced with the addition of oxLDL, indicating that higher oxLDL levels can further exacerbate cardiac adipogenesis.

To rescue the oxidative stress‐induced phenotype, the authors treated C‐MSC with the antioxidant, N‐acetyl cysteine (NAC), in the presence of adipogenic medium. NAC successfully reduced the increase in lipid content and reduced CD36 and PPARγ expression levels. These findings were substantiated through inhibition of CD36 or PPARγ in ACM C‐MSC leading to a reduction in lipid accumulation, further validating the involvement of the oxLDL/CD36/ PPARγ pathway in driving adipogenesis.

The authors corroborated their findings *in vivo* using the PKP2 heterozygous knockout mouse model. As ACM mouse models do not fully recapitulate all phenotypic aspects of the disease, especially the fibro‐fatty phenotype, the authors used a high‐fat diet approach to induce oxidative stress (McCauley & Wehrens, 2009). The high‐fat diet induced an increase in oxLDL levels, leading to increased fat accumulation in subepicardial areas and elevated protein expression of CD36 and PPARγ, accompanied with right ventricular dysfunction in PKP2 mutant mice compared to controls.

To test whether removing the burden of oxidative stress could alleviate fibro‐fatty progression in mice, the authors treated the high‐fat diet mice with atorvastatin, a frequently used drug to lower cholesterol levels in patients (Nissen *et al*, 2005). Compared to non‐treated PKP2 mutant mice, the atorvastatin‐treated group had lower oxLDL levels which also resulted in decreased expression of CD36 and PPARγ, in addition to reduced fat accumulation in the hearts (Fig 1). Moreover, the treatment ameliorated right ventricular function. This finding is of particular significance as these data suggest that lowering cholesterol levels might be of additional therapeutic benefit in ACM patients by halting fibro‐fatty replacement of the myocardium.

**Figure 1:**
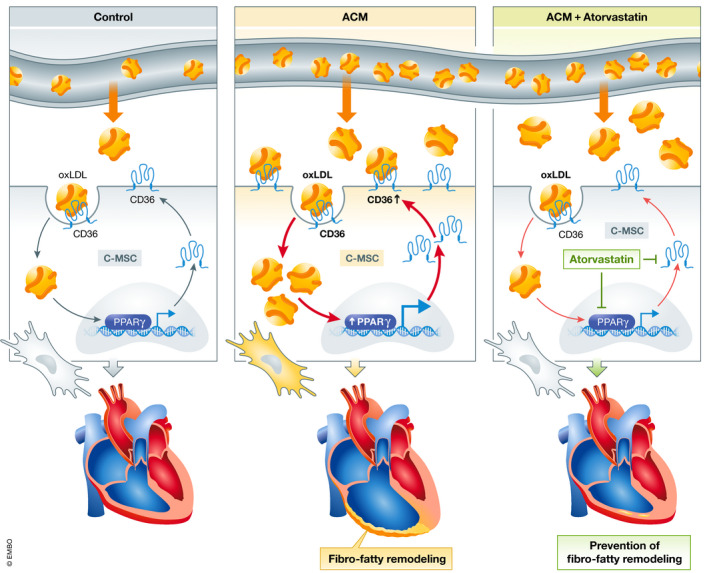
Inhibition of the oxLDL/CD36/ PPARγ axis by atorvastatin treatment prevents fibro‐fatty remodeling in mice A schematic showing high levels of circulating oxLDL in ACM resulting in increased uptake by C‐MSC through CD36, thus triggering activation of PPARγ. Augmented expression of CD36 consequently leads to a positive pathological feedback loop. Treatment with atorvastatin prevents fibro‐fatty remodeling through the reduction of CD36 and PPARγ levels. oxLDL, oxidized low‐density lipoprotein; ACM, arrhythmogenic cardiomyopathy; CD36, cluster of differentiation 36; C‐MSC, cardiac mesenchymal stromal cells; PPARγ, peroxisome proliferator‐activated receptor gamma. (Created with BioRender.com).

While previous studies have provided us with insights into triggers driving ACM, the exact biological mechanisms involved in the heterogeneous etiology of this pleiotropic disease are poorly characterized, especially related to fibro‐fatty replacement of the myocardium (Austin *et al*, 2019). By combining human patient data and relevant disease models, Sommariva *et al* (2021) show compelling evidence that oxidative stress is involved in adipogenesis in ACM. This study convincingly substantiates that treating oxidative stress with statins can impede fibro‐fatty development, thus improving heart function. The identification of higher levels of circulating cholesterol, thus oxLDL, as a novel risk factor opens an exciting new branch for potential future therapeutics for impeding the fibro‐fatty phenotype in ACM patients.
